# Efficacy of Specific Probiotic Strains in Subtypes of Irritable Bowel Syndrome: Systematic Review and Meta-Analysis of Randomized Controlled Trials

**DOI:** 10.3390/medicina62010089

**Published:** 2025-12-31

**Authors:** Abdulrahman Saud Almalki, Norah Yhya Jaafari, Norah Ghalib Aldossari, Anwar Ayed Alharbi, Abdulrahman Abdullah Alrdeeni, Alanoud Abdullah Alshareef, Abdulrahman Abed Al-subhi, Ammar Faisal Alsubhi, Muath Salem Alsubhi, Abdullah Almaqhawi

**Affiliations:** 1 College of Medicine, King Abdulaziz University, Jeddah 21589, Saudi Arabia; a.s.h.almalki111@gmail.com; 2 College of Medicine, Jazan University, Jazan 45142, Saudi Arabia; norahnorrr@gmail.com; 3 College of Medicine, King Faisal University, Al Hofuf 31982, Saudi Arabia; ng20m1g@gmail.com; 4 College of Medicine, King Abdulaziz University, Rabigh 25732, Saudi Arabia; aalsubhi0303@stu.kau.edu.sa (A.A.A.); aalrdeeni@stu.kau.edu.sa (A.A.A.); aalsubhi0261@stu.kau.edu.sa (A.A.A.-s.); aalsubhi0250@stu.kau.edu.sa (A.F.A.); malsobhi0080@stu.kau.edu.sa (M.S.A.); 5 Department of Biology, Faculty of Science, King Abdulaziz University, Jeddah 21589, Saudi Arabia; alanoudabdullah72@gmail.com; 6 Department of Family and Community Medicine, College of Medicine, King Faisal University, Al Hofuf 31982, Saudi Arabia

**Keywords:** irritable bowel syndrome, IBS subtypes, probiotics, specific probiotic strains, gut microbiota, randomized controlled trials, meta-analysis

## Abstract

*Background and Objectives*: The purpose of this systematic review and meta-analysis is to assess the patient’s quality of life, as well as the safety and effectiveness of different probiotic strains in treating IBS symptoms such as IBS-C, IBS-D, IBS-M, and IBS-U. Additionally, we contrast the side effects of probiotics that are single-strain and multi-strain. *Materials and Methods*: PRISMA criteria were followed in the conduct and reporting of this study. The protocol for this review (CRD420251120965) was entered into PROSPERO. RCTs comparing probiotic usage to placebo or usual therapy in adult IBS patients were found using four online databases: PubMed, Google Scholar, Ovid Medline, and the Cochrane Library. Review Manager was used for data synthesis and statistical analysis. *Results*: After screening 660 records, 16 randomized, double-blind, placebo-controlled trials with 2823 IBS patients were included. Probiotics significantly reduced intestinal discomfort overall (MD: −93.9; 95% CI −133.1 to −54.7; *p* < 0.00001) and the IBS-Severity Scoring System. Probiotics showed a clinically significant overall improvement (OR 1.71; 95% CI 1.26–2.33; *p* = 0.0006) when compared to a placebo. They were also well tolerated and did not increase adverse events. *Conclusions*: According to 16 RCTs, probiotics greatly lessen overall IBS symptoms, which enhances the quality of life and has global therapeutic implications. The findings support the use of probiotics as an effective and safe supplemental treatment for IBS patients.

## 1. Introduction

Irritable bowel syndrome (IBS) is a widely prevalent, chronic functional gastrointestinal disorder characterized by abdominal discomfort, including bloating, alterations in bowel habits, and recurrent abdominal pain [1]. This syndrome significantly affects patients’ health-related quality of life and imposes a substantial burden on healthcare systems worldwide [2]. Despite its high prevalence, the multifactorial etiology of IBS encompassing abnormalities in gastrointestinal motility, visceral hypersensitivity, alterations in gut microbiota, psychological comorbidities such as anxiety and depression, genetic predisposition, and sex-specific factors remains incompletely understood [3,4]. Central to the pathophysiology of IBS is a dysregulation of the gut–brain axis, which explains the frequent association of IBS with mood disorders and emphasizes the need for comprehensive, multidisciplinary management approaches [5].

Globally, the prevalence of IBS varies depending on the diagnostic criteria and population demographics. Some meta-analytical studies have indicated an overall prevalence of approximately 14.1%, with a female predominance (odds ratio 1.49) and psychological factors like stress playing significant contributory roles [6,7]. Regionally, prevalence estimates may differ, with rates reported as high as 20.7% in Saudi Arabia [8]. Diagnosis is primarily clinical, based on symptom criteria established by the Rome IV consensus, with IBS subtyped into four subgroups: constipation-predominant (IBS-C), diarrhea-predominant (IBS-D), mixed (IBS-M), and finally unclassified (IBS-U), which informs personalized management strategies [9,10].

Current treatment modalities, including dietary modification, pharmacotherapy, and behavioral interventions, are limited by suboptimal efficacy, variable patient response, and the risk of adverse effects [11,12]. Typically, dietary changes require careful supervision to prevent nutritional imbalances, as well as pharmacological agents that target isolated symptoms rather than addressing the overall manifestations of IBS [12]. All of these limitations necessitate exploration into alternative therapeutic modalities.

Probiotics, defined as live microorganisms which, when administered in adequate amounts, confer health benefits to the host, have gained interest as potential modulators of the gut microbiota aiming to restore microbial homeostasis, enhance mucosal barrier function, reduce inflammation, and modulate the gut–brain axis [13,14]. Specific probiotic strains, including *Bifidobacterium bifidum*, *Lactobacillus plantarum*, *Lactobacillus rhamnosus* GG, and *Bifidobacterium longum*, as well as multi-strain formulations, have demonstrated therapeutic potential, particularly in patients with moderate-to-severe IBS [14,15,16].

However, the wide heterogeneity in clinical trial designs, probiotic strains, dosages, administration durations, and outcome measures complicates the interpretation of efficacy [17,18,19,20,21,22,23]. Most importantly, the existing systematic reviews and meta-analyses often do not stratify effects according to IBS subtypes, and limited long-term safety data constrain the development of evidence-based guidelines [24,25]. This highlights a critical research gap concerning the identification of strain-, dose-, and subtype-specific probiotic efficacy and safety profiles.

Moreover, this systematic review and meta-analysis aim to rigorously evaluate the efficacy and safety of specific probiotic strains in managing distinct IBS subtypes, including IBS-C, IBS-D, IBS-M, and IBS-U, to establish evidence-based recommendations for personalized probiotic therapy. In line with this aim, the objectives of the review are to assess the effect of defined probiotic strains on global IBS symptom severity, including abdominal pain, bloating, and stool irregularities; evaluate the impact of probiotic interventions on IBS-related quality of life metrics; compare the therapeutic efficacy of single-strain versus multi-strain probiotic formulations; determine the influence of probiotic dosage and duration of treatment on clinical outcomes; synthesize safety data, including adverse events and treatment discontinuation rates associated with probiotic use in IBS patients; and explore subtype-specific responses to probiotics to identify optimal strain–subtype matching.

## 2. Materials and Methods

### 2.1. Registration and Protocol

This study was conducted and reported according to the Preferred Reporting Items (PRISMA) guidelines for systematic review and meta-analyses (Supplementary Materials Table S1) [26]. This review had been registered as a protocol on PROSPERO (CRD420251120965), which outlines our objective, methodology, and approach for our criteria [27]. Ethical approval and patient consent were not required as all the analyses were performed using previously published studies.

### 2.2. Search Strategy and Information Source

A literature review was conducted using four electronic databases as stated in our protocol: PubMed, Google Scholar, Web of Science, and Cochrane Library, with no restrictions applied to the publication date. The search strategy was constructed using the following key components: (“irritable bowel syndrome” OR “IBS” OR “spastic colon” OR “irritable colon” OR “functional bowel disorder”) AND (“probiotics” OR “probiotic therapy” OR “*Lactobacillus acidophilus*” OR “*Lactobacillus rhamnosus*” OR “*Lactobacillus casei*” OR “*Lactobacillus plantarum*” OR “*Lactobacillus reuteri*” OR “*Lactobacillus salivarius*” OR “*Bifidobacterium infantis*” OR “*Bifidobacterium longum*” OR “*Bifidobacterium breve*” OR “*Bifidobacterium bifidum*” OR “*Bifidobacterium lactis*” OR “*Saccharomyces boulardii*” OR “*Streptococcus thermophilus*” OR “*Enterococcus faecium*” OR “*Bacillus coagulans*” OR “*Escherichia coli* Nissle”) AND (“efficacy” OR “effectiveness” OR “outcomes”) AND (“randomized controlled trial” OR “RCT”). Furthermore, we extended our search by reviewing previously published systematic reviews and examining their reference lists to identify any RCTs that may have been missed during the initial literature search. We conducted the literature search and independently screened the retrieved articles for eligibility.

### 2.3. Eligibility Criteria

The studies included in our review met the following criteria for transparency and consistency with our protocols: they were randomized controlled trials (RCTs), controlled clinical trials, cohort studies (prospective or retrospective), or case-control studies. Participants were adults (≥16 years) diagnosed with IBS based on Rome II, III, or IV criteria. Each intervention involved clearly defined probiotic strains, used either as single-strain or multi-strain formulations, with a comparator group receiving placebo, standard care, or another specific probiotic formulation. Studies reporting clinical outcomes related to IBS symptoms, with a minimum duration of 4 weeks for probiotic intervention, were included, provided there was full-text availability in English. Studies were excluded if they were non-randomized, involved participants under 16 years of age, used undefined probiotic formulations, lacked a comparator group, had insufficient data, or were animal or in vitro studies, as were non-English publications.

### 2.4. Study Selection Process

The initial database search yielded 660 results. Following the title and abstract screening process, 195 duplicate entries were removed from the remaining 465 articles, resulting in 97 articles for further evaluation. The full-text review phase led to the selection of 97 eligible studies, which met the established inclusion criteria for data extraction and analysis. From these, 16 randomized controlled trials (RCTs) were selected for data extraction as they met the predefined criteria for inclusion.

### 2.5. Data Extraction and Outcome Measures

Two reviewers independently extracted data using a standardized form. The extracted data encompassed study characteristics (e.g., lead author, publication year, country, and study design), participant demographics, diagnostic criteria (Rome II, III or IV), probiotic treatment details (including strain, dosage, formulation, and duration), comparison groups, and documented IBS symptom outcomes. To ensure consistency in outcome measurement, only studies that assessed symptom severity using the IBS-Severity Scoring System (IBS-SSS) were pooled using the mean difference (MD). Studies employing different symptom scales were not combined with IBS-SSS outcomes. Any discrepancies between reviewers were resolved through mutual agreement.

### 2.6. Risk of Bias Assessment

Study quality was assessed using the Cochrane Risk of Bias (ROB 1) tool in RevMan 5.4, covering seven domains: random sequence generation, allocation concealment, blinding, incomplete outcome data, selective reporting, and other bias. Each domain was rated as having low, unclear, or high risk [28].

### 2.7. Statistical Analysis and Data Synthesis

Data synthesis and statistical analyses were performed using Review Manager (RevMan, Version 5.4; The Cochrane Collaboration, Oxford, UK) [29]. For continuous outcomes, pooled mean differences (MDs) and corresponding 95% confidence intervals (CIs) were calculated. MD was used only when all included studies within an outcome employed the same measurement scale (e.g., IBS-SSS). If future analyses require pooling studies using different scales, standardized mean difference (SMD) would be applied. For dichotomous outcomes, pooled odds ratios (ORs) with 95% CIs were estimated. A random-effects model (DerSimonian–Laird method) was applied when heterogeneity was substantial, while a fixed-effect model (Mantel–Haenszel method) was used for homogeneous data. Statistical heterogeneity among studies was assessed using the I^2^ statistic, with thresholds of 25%, 50%, and 75% representing low, moderate, and high heterogeneity, respectively. A *p*-value < 0.10 for the Chi-squared test indicated significant heterogeneity.

## 3. Results

### 3.1. Literature Search

**Figure 1:**
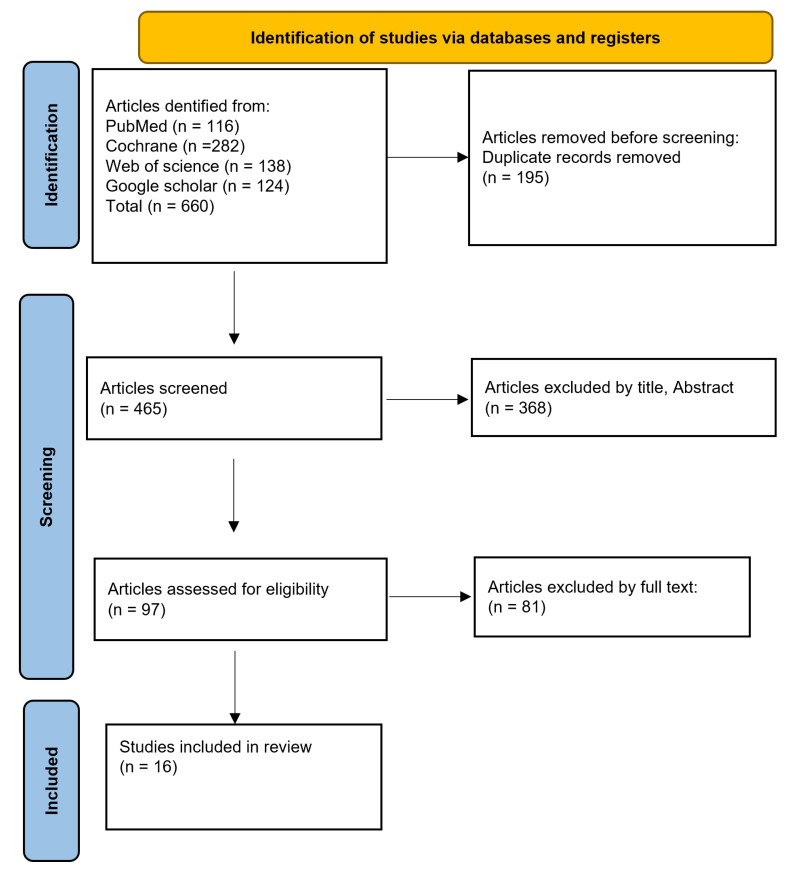
PRISMA 2020 flow diagram illustrating the selection process of studies included in the systematic review and meta-analysis (n = 16).

A total of 660 records were initially retrieved. After removing 195 duplicates, title and abstract screening excluded an additional 368 records. This process left 97 articles for full-text evaluation. Upon thorough assessment, 16 studies met all inclusion criteria and were incorporated into the final review (Figure 1).

### 3.2. Baseline Characteristics of Included Patients and Summary

Sixteen randomized, double-blind, placebo-controlled clinical trials were performed, encompassing 2823 patients diagnosed with irritable bowel syndrome (IBS). The study was conducted in Korea, Italy, India, Germany, and South Korea, mostly involving individuals aged 16 to 70 years. The IBS subtypes varied between trials, with most focusing on constipation-predominant (IBS-C) or diarrhea-predominant (IBS-D) forms, categorized according to Rome II, III or IV criteria. The number of male participants varied from 8 to 119 whilst female participants ranged from 18 to 317. All interventions involved the administration of oral probiotics, predominantly in capsule form, employing either single-strain or multi-strain formulations at daily dosages ranging from 5 × 10^9^ to 1 × 10^10^ CFU, administered once daily for a period of 4 to 8 weeks. The baseline demographic and clinical characteristics were comparable between the treatment and control groups across the studies, as illustrated in Table 1.

Based on our included randomized controlled trials, various probiotic strains exhibited notable effectiveness in mitigating symptoms of IBS, with differing impacts among IBS subtypes. *Lacticaseibacillus rhamnosus* IDCC 3201 and several multispecies formulations (e.g., *L. acidophilus*, *L. reuteri*, *L. plantarum*, *L. rhamnosus*, *B. lactis*) markedly alleviated IBS-C symptoms, especially abdominal bloating, pain, and bowel habit satisfaction, while improving the quality of life and modulating gut microbiota composition. In cases of IBS-D, various strains of *Lactiplantibacillus plantarum* (APsulloc 331261 GTB1, Jeju, Republic of Korea, CCFM8610 Jiangnan University Culture Collection Center (CCFM), Wuxi, China) and *Lactobacillus gasseri* BNR17 consistently alleviated symptom severity—particularly abdominal pain, distension, and diarrhea—while enhancing stool consistency, perceived stress, and quality of life, alongside beneficial alterations in microbiota and metabolic profiles. Bifidobacterium-based therapies (e.g., *B. longum* CECT 7347, *B. infantis* 35624, heat-inactivated *B. bifidum* MIMBb75) exhibited extensive symptom alleviation across IBS subtypes, enhancing abdominal pain, bloating, stool consistency, and anxiety, while being well tolerated. *Saccharomyces cerevisiae* CNCM I-3856 enhanced stomach discomfort and stool consistency across all subtypes, but one experiment indicated benefits mostly in IBS-C. In contrast, *L. plantarum* 299v, LCR35, and bacterial lysates produced variable or subtype-restricted advantages, as shown in Table 2.

### 3.3. Safety and Efficacy Outcomes

#### 3.3.1. IBS-Severity Scoring System (IBS-SSS)

**Figure 2:**
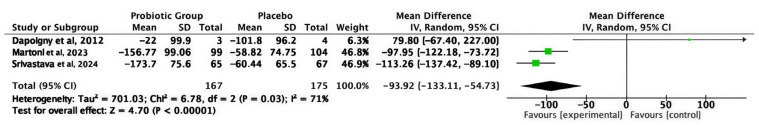
The IBS-Severity Scoring System (IBS-SSS) [34,35,41].

Pooled analysis of three studies showed that probiotics significantly decreased the IBS-SSS compared with placebo (MD: −93.92; 95% CI (−133.11 to −54.73); *p* < 0.00001), indicating a significant improvement in overall symptom severity. Pooled studies under the random effect model were heterogeneous (I^2^ = 71%), suggesting variation among probiotic strains or IBS subtypes. These results support a robust overall therapeutic effect of probiotics in alleviating IBS symptom burden, as shown in Figure 2.

#### 3.3.2. Intestinal Discomfort Symptoms

**Figure 3:**
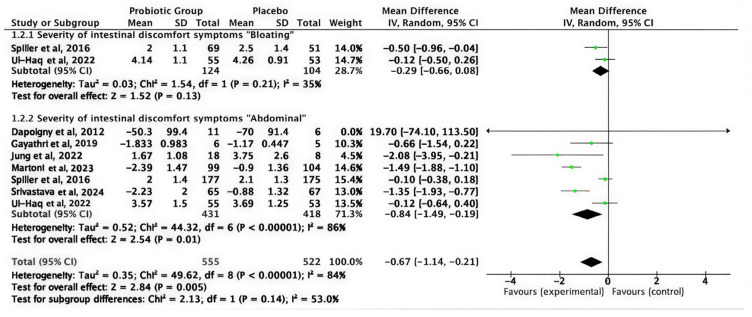
Intestinal discomfort symptoms [32,34,35,39,40,41,43].

A pooled analysis of nine studies evaluating distinct intestinal discomfort symptoms indicated that probiotics significantly alleviated overall intestinal discomfort compared to placebo (MD = −0.67; 95% CI (−1.14 to −0.21); *p* = 0.005). The pooled studies under the random effects model were heterogeneous (I^2^ = 84%). Subgroup analysis indicated a reduction in abdominal pain severity (MD = −0.84; 95% CI −1.49 to −0.19; *p* = 0.01; I^2^ = 86%), emphasizing that the greatest significant improvement was noted in pain-related symptoms, as shown in Figure 3.

All affected children in both groups. This difference was statistically significant (χ^2^ = 7.179, *p* = 0.008). The likelihood of enduring postoperative mild hearing loss was around 16 times greater in the mucoid group than in the serous group (adjusted OR = 15.97, 95% CI not displayed), indicating that mucoid effusions correlate with inferior auditory recovery post surgery.

#### 3.3.3. Frequency of Bowel Movement (Defecation Frequency)

**Figure 4:**
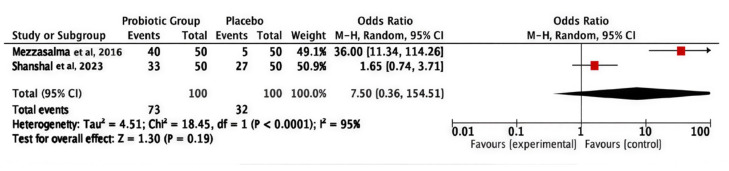
Frequency of bowel movement (defecation frequency) [31,37].

The pooled odds ratio was 7.50 (95% CI 0.36–154.51; *p* = 0.19), indicating a non-statistically significant difference between the two compared groups. Pooled studies under the random effect model were heterogeneous (I^2^ = 95%, *p* < 0.0001), predominantly influenced by Mezzasalma et al. (2016), which indicated a substantially greater effect (OR = 36.00; 95% CI 11.34–114.26) in contrast to Shanshal et al. (2023) (OR = 1.65; 95% CI 0.74–3.71), as shown in Figure 4 [31,37].

#### 3.3.4. Adequate Global Relief from IBS Symptoms

**Figure 5:**
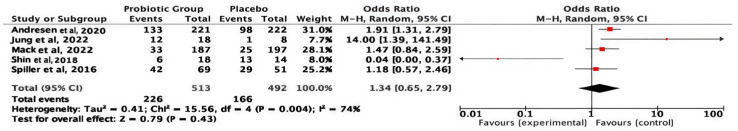
Adequate global relief from IBS symptoms [32,33,39,44,45].

The pooled analysis revealed no significant difference between probiotics and placebo (OR = 1.34, 95% CI 0.65–2.79; *p* = 0.43), suggesting variability in response across strains and subtypes, as shown in Figure 5. Pooled studies under the random effect model were heterogeneous (I^2^ = 74%, *p* = 0.004). This inconsistency was likely influenced by the heterogeneous definitions used for “adequate global relief” across trials. Some studies assessed weekly symptom resolution whereas others used multi-week or composite criteria, leading to nonuniform thresholds for defining clinical improvement. These methodological differences may partially explain the discrepancy between this outcome and the more consistent findings presented in Figure 2 and Figure 3. Standardized definitions of global relief will be essential to improve comparability in future research.

#### 3.3.5. Quality of Life (QoL)

**Figure 6:**
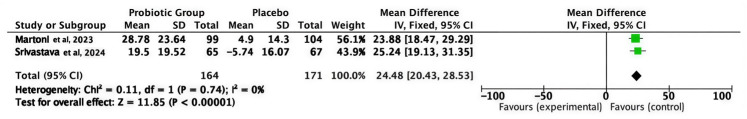
Quality of life (QoL) [34,35].

Pooled analysis indicated that probiotics significantly enhanced the quality of life for individuals with IBS (MD = 24.48; 95% CI 20.4–28.5; *p* < 0.00001). Because higher QoL scores indicate better patient well-being, the positive mean difference reflects a greater improvement in the probiotic group. Although the forest plot visually places the diamond toward the “control” side due to scale orientation, the numerical results consistently favor probiotics. Pooled studies under the fixed effect model were homogeneous (I^2^ = 0%, *p* = 0.7), as shown in Figure 6.

#### 3.3.6. Stool Type Classification (Bristol Stool Scale)

**Figure 7:**
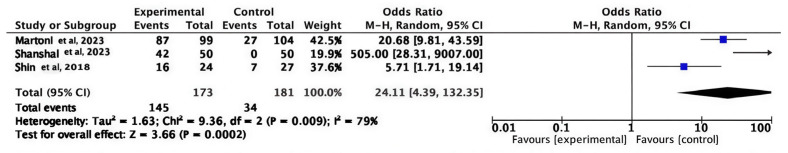
Stool type classification (Bristol Stool Scale) [33,34,37].

A pooled analysis of three studies indicated that probiotics enhanced stool consistency relative to placebo (OR = 24.11; 95% CI 4.39–132.3; *p* = 0.0002). Pooled studies under the random effect model were heterogeneous (I^2^ = 79%), attributed to variations in probiotic formulations and IBS subtypes, as illustrated in Figure 7.

#### 3.3.7. FDA-Defined Global Improvement (Responder Analysis)

**Figure 8:**
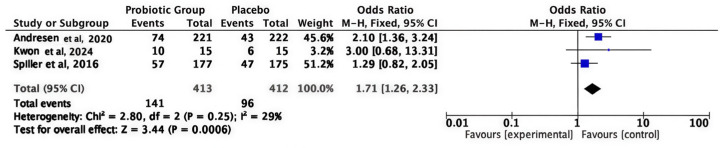
FDA-defined global improvement (responder analysis) [30,39,44].

The pooled analysis of three studies showed a clinically meaningful improvement in overall IBS symptoms consistent with FDA responder definitions and the superiority of probiotics over placebo (OR = 1.71; 95% CI 1.26–2.33; *p* = 0.0006). Pooled studies under the fixed effect model were homogeneous (I^2^ = 29%), as shown in Figure 8.

#### 3.3.8. Adverse Events

**Figure 9:**
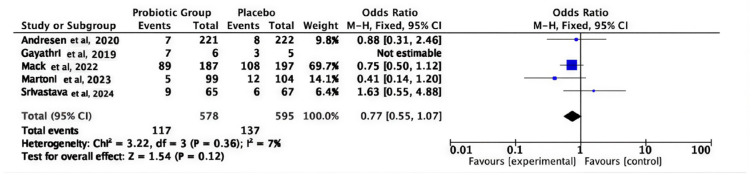
Adverse events [34,35,40,44,45].

The pooled analysis revealed that the incidence of adverse events was comparable between the two groups (OR = 0.77; 95% CI 0.55–1.07; *p* = 0.12); the pooled studies under the fixed effect model demonstrated homogeneity (I^2^ = 7%), suggesting that probiotics were predominantly well tolerated without an elevated risk of adverse effects, as shown in Figure 9.

### 3.4. Risk of Bias Assessment

**Figure 10:**
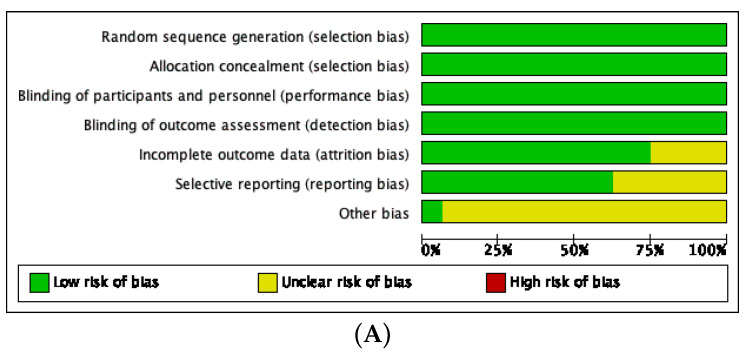

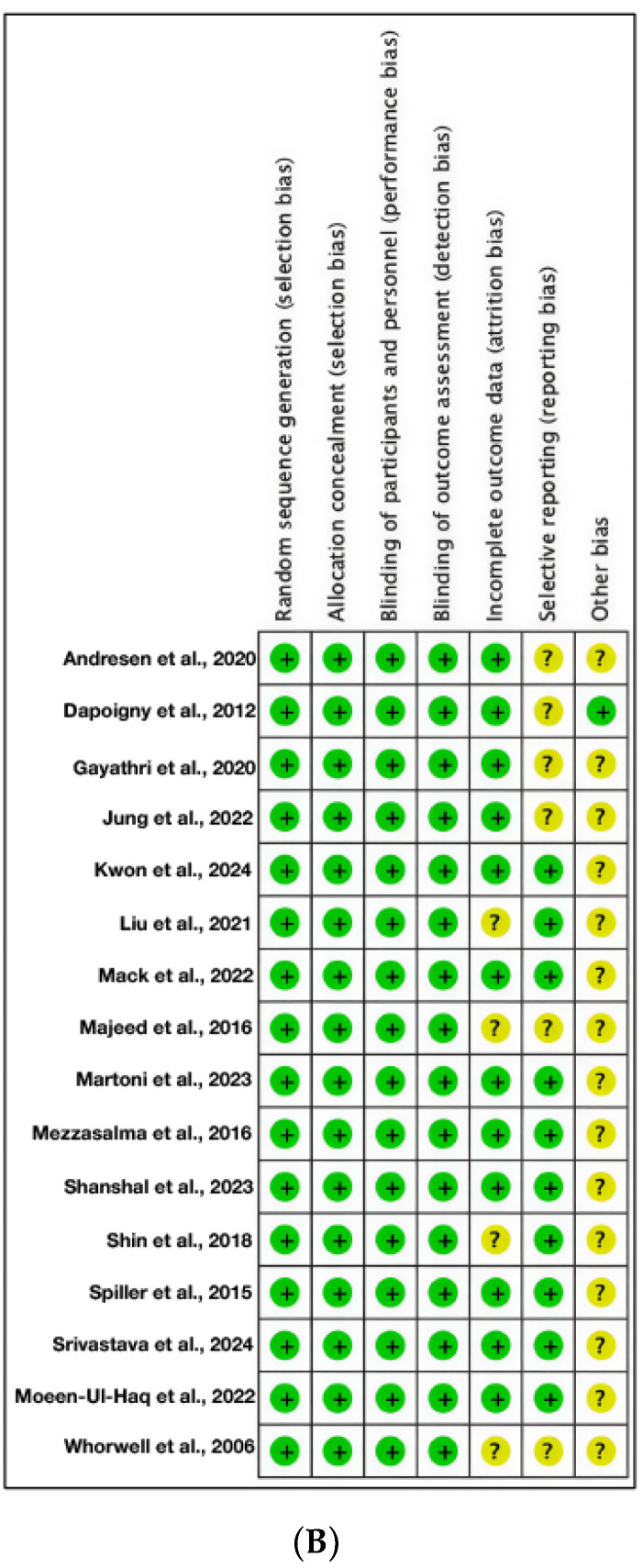
(**A**) Risk of bias assessment summary graph. (**B**) Risk of bias assessment [30,31,32,33,34,35,36,37,38,39,40,41,42,43,44,45].

The risk-of-bias evaluation revealed that most studies demonstrated a low risk of bias across all domains, particularly in randomization, allocation concealment, and blinding. A few studies showed an unclear risk, mainly due to incomplete outcome data, selective reporting, or other potential biases, while no high-risk assessments were identified, as illustrated in Figure 10A,B.

## 4. Discussion

This systematic review with meta-analysis combines results from 16 well-conducted randomized controlled trials (RCTs) including 2823 patients with irritable bowel syndrome (IBS). The findings provide strong evidence supporting the effectiveness of oral probiotics in achieving IBS symptom control. The primary outcome demonstrates that probiotics are significantly more beneficial than placebo in improving overall symptoms, with a substantial reduction in IBS-Severity Scoring System (IBS-SSS) scores (mean difference = −93.92; 95% CI: −133.11 to −54.73; *p* < 0.00001). This improvement suggests a meaningful enhancement in patient quality of life. Consistent gains in global improvement—based on FDA-proposed responder definitions (OR = 1.71; 95% CI: 1.26–2.33; *p* = 0.0006)—and low heterogeneity (I^2^ = 29%) further reinforce the reliability of these results. Additionally, reductions in abdominal discomfort support potential mechanisms involving gut–brain axis modulation, visceral sensitivity, or microbiome regulation [46].

The results align with previous evidence supporting probiotic use in IBS [47]. The favorable safety profile (OR = 0.77; *p* = 0.12; I^2^ = 7%) suggests no increased risk of adverse events compared with placebo. However, interpreting continuous outcomes remains challenging due to high heterogeneity in IBS-SSS (I^2^ = 71%) and intestinal discomfort (I^2^ = 84%). This variability may reflect clinical diversity related to heterogeneous IBS subtypes [48] and the strain-specific nature of probiotic effects [49]. Methodological differences including variable strain combinations, dosages, and formulations also contribute to inconsistencies in reported efficacy [50].

Current clinical guidelines provide a nuanced context for interpreting these findings. The 2021 American College of Gastroenterology (ACG) Clinical Guideline for the Management of IBS suggests against the routine use of probiotics for global IBS symptoms, citing low certainty of evidence and substantial heterogeneity across trials [51]. This cautious recommendation reflects earlier inconsistencies in probiotic formulations, strain selection, and outcome reporting rather than a clear lack of therapeutic effect. Importantly, the present meta-analysis incorporates a larger and more contemporary evidence base, including higher-quality RCTs and standardized outcome measures, which may partially address the limitations highlighted in the ACG guideline. Our findings therefore contribute updated evidence that may inform future guideline revisions, particularly as probiotic research continues to evolve.

Recent evidence highlights the potential superiority of multi-strain and multispecies probiotic formulations. For example, Allegretti et al. (2025) reported significant improvements in bloating, gas, and abdominal discomfort using a 24-strain synbiotic, suggesting that broader microbial diversity may enhance clinical benefits [52]. These advanced diagnostic tools allow clinicians to characterize individual microbiome patterns before and after treatment, offering opportunities to tailor probiotic interventions. With microbiome assessment becoming increasingly integrated into clinical care, these approaches may refine future therapeutic strategies [53]. A recent meta-analysis by Yang et al. (2024), which included 72 RCTs, demonstrated modest but significant improvements in global IBS symptoms and abdominal pain with probiotic use while similarly noting considerable heterogeneity and strain-specific effects [15].

The clinical implications of this review are noteworthy. Probiotics demonstrate measurable symptom relief, good tolerability, and potential value for patients who do not respond adequately to first-line therapies. These findings support considering probiotics as a meaningful component of IBS management rather than solely an adjunctive option. At the same time, the growing evidence base for multi-strain and personalized probiotic therapy underscores the need for updated clinical frameworks that account for strain specificity, microbiome profiles, and biological mechanisms.

Future research should focus on large, well-designed RCTs evaluating specific strains in defined IBS subtypes; mechanistic studies addressing microbial changes, SCFA pathways, and mucosal barrier effects; and long-term follow-up to understand sustained benefits. Despite some limitations, including heterogeneity and incomplete reporting, this review provides robust evidence supporting probiotic use while highlighting the need for more precise and personalized therapeutic approaches.

## 5. Conclusions

This systematic review and meta-analysis study of 16 randomized controlled trials involving 2823 patients with irritable bowel syndrome provides robust evidence supporting the efficacy of oral probiotics for symptom management. The results demonstrated that probiotic therapy significantly reduced total symptom severity and meaningful global improvement compared with placebo, with additional benefits observed across key symptom domains (abdominal pain and diarrhea). Importantly, probiotics demonstrated a favorable safety profile, with no significant increase in adverse events relative to placebo.

Taken together, all of these findings indicate that probiotics represent a safe and effective therapeutic option for improving symptoms in overall patients who have IBS. However, given the variability in probiotic strains, dosages, and study durations, further well-designed, large-scale trials are warranted to establish optimal regimens and confirm long-term benefits.

## Figures and Tables

**Table 1:** Baseline characteristics of participants. Baseline characteristics of 2823 participants from sixteen RCTs are summarized. Most studies included IBS-D or IBS-C patients diagnosed using Rome II, III or IV criteria, with similar baseline profiles between groups.

Study ID	Sample Size	Age Range	IBS Subtype (IBS-C, IBS-D, IBS-M, IBS-U)	Diagnostic Criteria	Males	Females
Kwon et al., 2024 [30]	30	NM	IBS-C (constipation-predominant IBS)	Rome IV	NM	NM
Mezzasalma et al., 2016 [31]	150	18–65 years	IBS-C (constipation-predominant IBS)	Rome III	NM	NM
Jung et al., 2022 [32]	27	>19 years	Diarrhea-predominant IBS (IBS-D)	Rome IV	8	18
Shin et al., 2018 [33]	60	20–55 years	Diarrhea-predominant IBS (IBS-D)	Rome III	22	29
Martoni et al., 2023 [34]	307	18–70 years	Diarrhea-predominant IBS (IBS-D)	Rome IV	NM	NM
Srivastava et al., 2024 [35]	200	18–65 years	Diarrhea-predominant IBS (IBS-D)	Rome IV	127	73
Majeed et al., 2016 [36]	36	18 to 55 years	Diarrhea-predominant IBS (IBS-D)	Rome III	17	19
Shanshal et al., 2023 [37]	100	16–55 years	Diarrhea-predominant IBS (IBS-D)	Rome IV	53	45
Liu et al., 2021 [38]	75	27–79 years	Diarrhea-predominant IBS (IBS-D)	Rome III	32	43
Spiller et al., 2015 [39]	379	18–75 years	IBS-D, IBS-C, IBS-M, IBS-U	Rome III	62	317
Gayathri et al., 2020 [40]	100	≥18 years	IBS-D, IBS-C, IBS-M, IBS-U	Rome III	66	34
Dapoigny et al., 2012 [41]	50	18–70 years	IBS-D, IBS-C, IBS-M, IBS-U	Rome III	15	35
Whorwell et al., 2006 [42]	362	18–65 years	IBS-D, IBS-C, IBS-M, IBS-U	Rome II	NM	NM
Moeen-Ul-Haq et al., 2022 [43]	120	Group A (*Lactobacillus plantarum* 299v): Mean age is 37.53 ± 9.02 years. Group B (Placebo): Mean age is 34.40 ± 11.23 years.	IBS-D, IBS-C, IBS-M, IBS-U	Rome III	63	45
Andresen et al., 2020 [44]	443	≥18 years	IBS-D, IBS-C, IBS-M, IBS-U	Rome III	NM	NM
Mack et al., 2022 [45]	384	≥18 years	IBS-D, IBS-C, IBS-M, IBS-U	Rome III	119	265

**Table 2:** Summary of study design and intervention characteristics. This summarizes the key methodological and intervention features of the sixteen included trials, detailing study design, country, probiotic formulation, dosage, administration route, and treatment duration.

Study ID	Study Design	Country /Origin	Formulation (Single-Strain vs. Multi-Strain)	Administration Route	Dosage (CFU/Day)	Frequency	Duration of Treatment (Weeks)	Findings
Kwon et al., 2024 [30]	Randomized, double-blind, placebo-controlled trial	Korea	Single-strain	Capsule	1 × 10^10^ CFU/day	Once daily	8 weeks	*Lacticaseibacillus rhamnosus* IDCC 3201 significantly improved IBS-C symptoms including abdominal bloating, bowel habits, and quality of life, meeting FDA responder criteria. This was associated with favorable changes in gut microbiota (enrichment of *A. muciniphila* and *B. cellulosilyticus*) and specific fecal metabolites.
Mezzasalma et al., 2016 [31]	Randomized, double-blind, placebo-controlled trial	Italy	Multi-strain	Capsule	5 × 10^9^ CFU each strain	Once daily	60 days (~8.5 weeks)	Multispecies probiotic formulations (G1: *L. acidophilus*, *L. reuteri*; G2: *L. plantarum*, *L. rhamnosus*, *B. animalis* subsp. *Lactis*) significantly improved IBS-C symptoms (like bloating, pain, and constipation) and enhanced health-related quality of life compared to placebo, with effects largely maintained after treatment cessation. The probiotics were successfully detected in the gut, showing favorable changes in the fecal microbiota.
Jung et al., 2022 [32]	Randomized, double-blind, placebo-controlled trial	South Korea	Single-strain	capsule	1 × 10^10^ CFU/day	Once daily	4 weeks + 2-week follow-up	*Lactiplantibacillus plantarum* apsulloc 331261 (GTB1) significantly improved global relief from IBS-D symptoms, reduced abdominal pain and bloating severity/frequency, decreased diarrhea frequency, and enhanced quality of life by modulating fecal microbiota, with no significant adverse events.
Shin et al., 2018 [33]	Randomized, double-blind, placebo-controlled trial	Korea	Single-strain	Capsule	10^10^ CFU/day	4 capsules per day (2 after breakfast and 2 after dinner)	8 weeks	*Lactobacillus gasseri* BNR17 significantly improved IBS-D symptoms including abdominal pain, distension, and diarrhea, along with quality of life, by increasing colon transit time and favorably altering gut microbiota, while also reducing fasting blood glucose, all with minimal adverse events.
Martoni et al., 2023 [34]	Randomized, double-blind, placebo-controlled trial; multi-center and dose-ranging study	Denmark	Multi-strain	Capsule	Two doses were used: 1 × 10^9^ (1B) colony-forming units/d and 1 × 10^10^ (10B) colony-forming units/d.	One capsule daily before lunch.	8 weeks	The study found that *Lactiplantibacillus plantarum* Lpla33 significantly reduced overall IBS symptom severity and normalized bowel habits in adults with diarrhea-predominant IBS, with a dose-ranging effect observed for the higher dose. The probiotic was also well tolerated and improved quality of life and perceived stress.
Srivastava et al., 2024 [35]	Randomized, double-blind, placebo-controlled trial	India	Multi-strain	Capsule	1 × 10^9^ colony-forming units/day; HT-ES1: 2.5 × 10^9^ cells/day (equivalent to 50 mg/day).	Once daily	12 weeks	The study found that *Bifidobacterium longum* CECT 7347 (ES1, probiotic) and its heat-treated form (HT-ES1, postbiotic) significantly reduced overall IBS symptom severity, abdominal pain, and anxiety while improving quality of life and stool consistency in adults with diarrhea-predominant IBS. Both forms were well tolerated and effective.
Majeed et al., 2016 [36]	Randomized, double-blind, placebo-controlled, multi-centered trial.	India	Maltodextrin equivalent to the active tablet.	Oral, one tablet daily	None (placebo)	Once daily, 30 min before a meal.	90 days.	The study concluded that the *B. coagulans* MTCC 5856 at a dose of 2 × 10^9^ CFU/day along with standard care of treatment was found to be safe and effective in diarrhea predominant IBS patients for 90 days of supplementation. Hence, *B. coagulans* MTCC 5856 could be a potential agent in the management of diarrhea predominant IBS patients.
Shanshal et al., 2023 [37]	Randomized, single-blind, placebo-controlled trial	Iraq	Multi-strain	capsule	2 × 10^9^ CFU	twice daily	12 weeks	Adding *L. acidophilus* and *L. plantarum* probiotics to standard treatment significantly reduced overall IBS-D symptom severity and its components (abdominal pain, distension, bowel habit satisfaction, and life interference) and improved IBS severity classification, outperforming standard treatment alone. The probiotics also showed a marked effect on reducing mucus in stool and urgency.
Liu et al., 2021 [38]	Randomized, double-blind, placebo-controlled trial	China	Single-strain	Powder	1 × 10^10^ CFU/day	Once daily	8 weeks	*Lactobacillus plantarum* CCFM8610 significantly alleviated IBS-D clinical symptoms (reducing severity and bloating and improving bowel habit satisfaction), enhanced quality of life, and reversed gut microbiota dysbiosis by increasing beneficial butyrate-producing bacteria and decreasing bloating-related Methanobrevibacter, all while being well tolerated and safe.
Spiller et al., 2015 [39]	Randomized, double-blind, placebo-controlled trial; multi-center	France	Single-strain	Capsule	1000 mg per day (equivalent to 8 × 10^9^ colony forming units (CFU)/g)	Once daily	12 weeks	The study found no overall benefit of *Saccharomyces cerevisiae* I-3856 for general IBS symptoms. However, it demonstrated a significant improvement in abdominal pain/discomfort and bloating in the IBS-C subgroup compared to placebo.
Gayathri et al., 2020 [40]	Randomized, single-blind, placebo-controlled trial	India	Single-strain	capsule	2 × 10^9^ CFU	Twice daily	8 weeks	The study demonstrated that *Saccharomyces cerevisiae* CNCM I-3856 significantly reduced abdominal pain and improved stool consistency across all IBS subgroups (IBS-C, IBS-D, IBS-M) compared to placebo. It was well tolerated with no serious adverse events.
Dapoigny et al., 2012 [41]	Randomized, double-blind, placebo-controlled trial; pilot study	France	Single-strain	capsule	2 × 10^8^ CFU	Once daily (3 capsules together in fasting state)	4 weeks	The results indicated that LCR35 did not produce a statistically significant improvement in global IBS symptoms. Nonetheless, within the IBS-D subgroup, LCR35 was associated with meaningful decreases in overall symptom intensity and abdominal pain, showed a greater proportion of treatment responders, and demonstrated good tolerability across all subgroups.
Whorwell et al., 2006 [42]	Randomized, double-blind, placebo-controlled, multi-center trial	United Kingdom	single-strain	capsule	1 × 10^6^, 1 × 10^8^, or 1 × 10^10^ CFU per dose	Once daily	4 weeks	The study found that *Bifidobacterium infantis* 35624 at a dose of 1 × 10^8^ CFU significantly relieved multiple IBS symptoms, including abdominal pain and bloating, and improved overall IBS relief and bowel habit satisfaction in women with IBS. It also normalized bowel movement frequency at the extremes and was well tolerated across IBS-C, IBS-D, and IBS-A subgroups.
Moeen-Ul-Haq et al., 2022 [43]	Randomized controlled trial (RCT)	Pakistan	Single-strain	Capsule	5 × 10^10^ CFU	Once daily	4 weeks	The study concluded no significant overall efficacy of *Lactobacillus plantarum* 299v in improving IBS symptoms compared to placebo. However, sub-analysis showed significant reduction in abdominal pain severity for IBS-C and IBS-D subgroups at the end of therapy with *L. plantarum* 299v.
Andresen et al., 2020 [44]	A multi-center, randomized, double-blind, placebo-controlled clinical trial	Germany	Multi-strain	Capsule	1 × 10^9^ non-viable *B. bifidum* HI-MIMBb75 cells per day. Two capsules were taken orally once a day	Once a day	8 weeks	The study demonstrated that heat-inactivated *Bifidobacterium bifidum* mimbb75 (SYN-HI-001) significantly improved overall IBS symptoms, including abdominal pain, bloating, and global relief, compared to placebo. It also showed subtype-specific benefits, such as increased bowel movements in IBS-C and improved stool consistency in IBS-D, and was well tolerated.
Mack et al., 2022 [45]	A phase IV, randomized, double-blind, placebo-controlled, multi-center (30 study sites), parallel group study	Germany	Multi-strain	Oral drops	1.5-4.5 × 10^7^ *E. coli* and 1.5-4.5 × 10^7^ *E. faecalis* per mL. The dosage schedule was 10 drops (0.71 mL) 3×/day during week one, 20 drops (1.42 mL) 3×/day during week two, and 30 drops (2.14 mL) 3×/day during week three, with maintenance dosing of 30 drops 3×/day.	Three times a day	26 weeks	The study found that a bacterial lysate was not effective across all IBS subtypes for primary global or abdominal pain endpoints. However, post hoc and sensitivity analyses suggested potential benefits in the IBS-D subgroup, showing a trend for improved abdominal pain response, reduction in diarrhea, and a higher responder rate (IBS-AP75). The treatment was well tolerated.

## Data Availability

The data generated in this study are available upon reasonable request from the corresponding author.

## Supplementary Materials

The following supporting information can be downloaded at: https://www.mdpi.com/article/10.3390/medicina62010089/s1, The PRISMA 2020 checklist.
